# SAFE: an eHealth intervention for women experiencing intimate partner violence – study protocol for a randomized controlled trial, process evaluation and open feasibility study

**DOI:** 10.1186/s12889-020-08743-0

**Published:** 2020-05-07

**Authors:** N. E. van Gelder, K. A. W. L. van Rosmalen-Nooijens, S. A Ligthart, J. B. Prins, S. Oertelt-Prigione, A. L. M. Lagro-Janssen

**Affiliations:** Department of Gender in Primary and Transmural Care, Radboud University Medical Center, Postbus 9101, 6500 HB Nijmegen; Geert Grooteplein 21 – route 117, 6525 EZ Nijmegen, the Netherlands

**Keywords:** Ehealth, E-health, women’s health, Online intervention, Intimate partner violence, IPV, Domestic violence, DV, Randomized controlled trial, Process evaluation

## Abstract

**Background:**

Intimate partner violence (IPV) affects almost one in three women worldwide. However, disclosing violence or seeking help is difficult for affected women. eHealth may represent an effective alternative to the standard support offers, which often require face-to-face interaction, because of easy accessibility and possibility of anonymous usage. In the Netherlands we are developing SAFE, an eHealth intervention for female victims of IPV, which will be evaluated in a randomized controlled trial and a process evaluation, followed by an open feasibility study to assess real-world user data.

**Methods/design:**

The randomized controlled trial is a two-arm parallel design comparing an intervention arm and a control group. The groups both have access to eHealth but differ in the offer of interactive features compared to static information. Both groups complete questionnaires at three or four time points (baseline, three months, six months, 12 months) with self-efficacy at 6 months as the primary outcome, measured with the General Self-Efficacy (GSE) scale. The process evaluation consists of quantitative data (from the website and from web evaluation questionnaires) and qualitative data (from interviews) on how the website was used and the users’ experiences.

**Discussion:**

eHealth has the potential to reach a large number of women who experience IPV. The internet-based design can lower access barriers and encourage help-seeking behavior ultimately reducing the lag time between subjective awareness and protective action.

**Trial registration:**

Trial registered on 15 August 2017 at the Netherlands Trial Register NL7108 (NTR7313).

## Background

Intimate partner violence (IPV) is any physical, sexual, psychological, or economic violence that occurs between former or current partners [1]. While both men and women can suffer from IPV, the majority of victims are female. Worldwide almost one in three women experience IPV in their lifetime [1–3]. Being a victim of IPV has numerous negative consequences at the physical, social and psychological level. For example, a higher risk of developing depression, anxiety disorders, and post-traumatic stress syndrome (PTSS) [4, 5]. In addition, children exposed to IPV are at increased risk for developing trauma and are three times more likely to become a perpetrator or a victim of violence themselves [6–11].

Women report major obstacles in disclosing IPV and seeking help. Delays in help-seeking are related to fear, shame, guilt, loyalty, children, financial dependency, not knowing where to go for help, and not subjectively identifying IPV as such. Attempts to overcome these barriers have had limited effects [12–19]. Even though the governmental and political efforts to decrease IPV and its detrimental effects have substantially increased over the last decades, there is still a need for effective interventions that can reduce the lag time between exposure and active help-seeking.

eHealth is becoming more popular in healthcare [20–22]. For potentially stigmatizing and traumatizing experiences, such as IPV, eHealth represents an ideal option because it is easily accessible, allows anonymity and thereby safety and does not require face-to-face contact [23]. It has the potential of reaching large numbers of women exposed to IPV. Some eHealth offers for women exposed to IPV have already been reported outside of Europe: I-DECIDE in Australia [24], *isafe* in New-Zealand [25], IRIS in the United States [26], and iCAN in Canada [27]. These interventions are completely online based, and all provide a (personalized) safety plan for participants and information on help for IPV. To our knowledge, no such intervention has been developed in Europe to date, although the prevalence rates do not differ from the rest of the world [3]. To fill this gap, we developed an eHealth intervention in the Netherlands, SAFE, to be found at www.safewomen.nl. SAFE was inspired by I-DECIDE [24] and Feel the ViBe, a Dutch internet intervention for youth exposed to family violence [23]. Furthermore, SAFE is based on available scientific knowledge and a national qualitative study on experiences from female survivors and IPV experts (Gelder et al., forthcoming). SAFE will be evaluated through a randomized controlled trial, a process evaluation, and an open feasibility study.

### Aims of SAFE: effectiveness, process evaluation and feasibility

The SAFE randomized controlled trial (RCT) is designed to evaluate the effectiveness of an interactive intervention in increasing self-efficacy at six months in women exposed to IPV, compared to a minimal intervention. The secondary outcomes assessed are self-awareness, (mental) health symptoms and perceived support in women exposed to IPV.

The primary aim of the process evaluation is to evaluate the feasibility of SAFE with the following research question:

Is SAFE a suitable tool to provide information and support to women exposed to IPV?

Firstly, we will discuss the study procedure of the RCT. Subsequently the process evaluation and the open feasibility study are described, followed by the discussion.

## RCT methods and design

The RCT investigates two groups: the SAFE group, which receives the complete intervention, and the control group, receiving a minimal intervention as described below. The RCT has a parallel design with randomization balanced on age (18–30 years old and 31–50 years old). The study is conducted in the Netherlands and the intervention is only available in Dutch. The trial is registered at the Netherlands Trial Register (NTR) with trial ID NTR7313. The protocol is described conforming to the CONSORT EHEALTH guidelines [28].

### Sample size calculation

The sample size was calculated based on the primary outcome measure, the General Self-Efficacy Scale (GSE). In the general population, a mean score of 29.3 (SD 4.6) was described [29, 30]. We measure GSE scores at T0 (baseline), T1 (three months), T2 (six months), and T3 (12 months). We consider a difference of at least two points relevant and to detect this difference, 85 participants will be needed (power = 80%, alpha = 5% two-sided testing) in each group at T2 (six months). If we apply an ANCOVA analysis with the T2 measurement as a dependent variable and correct for the T0 measurement, the sample size has to be adjusted with the factor (1 - r^2^) (89). With r as the correlation between T0 and T2. We use *r* = 0.5 which translates into 64 participants in each group. We assume a relatively high attrition rate (35%) at T3. Such an attrition rate is common in eHealth research and similar designs [23, 26, 27, 31–33]. Therefore, we plan to include 99 participants in each group (198 participants in total) at baseline, with an observation period of six till 12 months. Participants included during the period April 2019 – March 2020 will be followed for 12 months, completing surveys at four time points (T0 – T3). Participants included in the extended inclusion period, due to high attrition and low response rates, April 2020 – October 2020, will be followed for six months and completing surveys at three time points (T0 – T2) in order to not interfere with the planned open feasibility study.

### Inclusion and exclusion criteria

Inclusion criteria:

- identifying as woman;
- 18–50 years old;
- self-identifying as a victim of IPV;
- having access to a computer and internet connection.

Exclusion criteria:

- reporting no unhealthy or abusive relationship or experienced fear of partner in the past 12 months at T0;
- inability to read and understand the Dutch language (all outcome measures are in Dutch);

However, women who are over 50 or who experienced IPV or fear of partner longer than 12 months ago are given access to the control group version of the website. No data will be collected from the excluded participants.

### Recruitment, inclusion and randomization

Participants are mostly recruited online, through social media, Facebook Ads and Google Ads. Several (mental) health organizations (e.g. general practitioners’ offices, emergency rooms, physiotherapists, social workers) are contacted with information about SAFE and provided with a (digital) poster that can be shown in the waiting room.

**Fig. 1:**
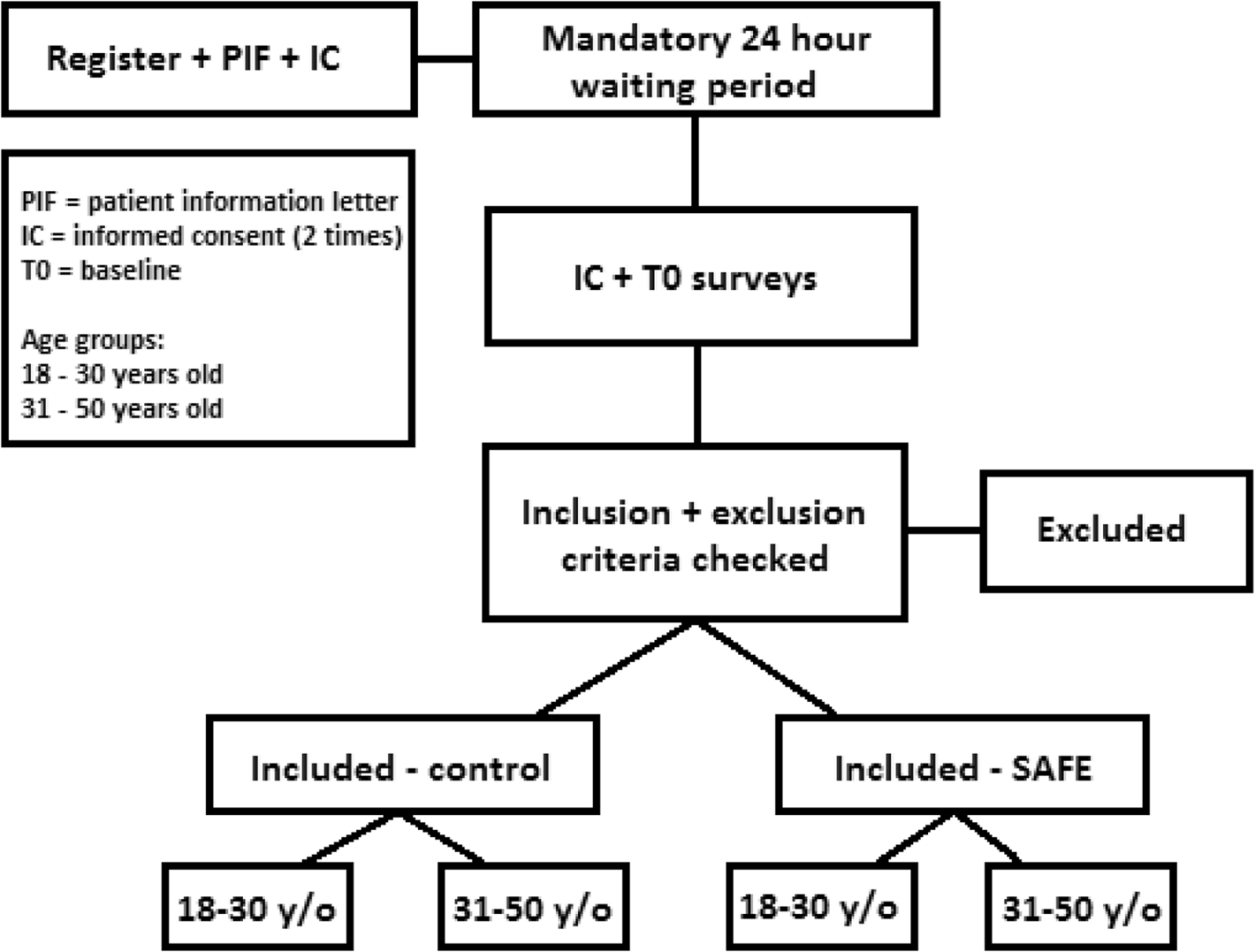
Online inclusion procedure

Women interested in using SAFE must register online and give consent to participate in the study. Women read the patient information letter and check a box to give consent. This is followed by a mandatory 24-h waiting period to ensure participants had sufficient time to contemplate participating in the study. After 24 h they are asked to give consent for a second time and fill out the T0 questionnaires. Subsequently, after checking inclusion criteria, they are randomized automatically. A stratified (block size of four) randomization balancing for age will be performed (Fig. 1). This random allocation sequence with age blocks is generated by the eHealth developer and a statistician. Randomization is single-blind to the participants but not to the researchers. Participants have immediate access to the website after completing the T0 questionnaires.

### Intervention

#### Development

In the development phase we interviewed 16 women to explore wishes and needs as key elements for SAFE. The women were survivors of and/or experts on domestic violence (DV) and IPV. We also assessed women’s needs concerning their safety when using SAFE (Gelder et al. forthcoming). With this input, together with other (scientific) sources such as the I-DECIDE study [24], we developed a prototype, which was tested in a focus group of previously interviewed women. The prototype was improved based on the feedback and tested again in a focus group, resulting in a definitive version of the intervention used in this RCT.

#### Structure of the intervention

The SAFE intervention contains various modules and means of contact. The full intervention consists of four modules (Table 1). The modules provide information, tips, and exercises. Information that SAFE provides is based on scientific research and on information provided by IPV and DV help or research organizations.

**Table 1:** SAFE modules and functionalities

***My situation (module)***	***Help (module)***
*Intimate partner violence (IPV)*	*Help options*
*Relationships (healthy and unhealthy)*	*Help database*
*Impact on children*	*Safety*
**My health (module)**	**My environment (module)**
Physical health	Social support
Mental health	Contact
Symptoms and tips	Disclosing IPV
***Contact***	***About SAFE***
*Contact options with fellow survivors and victims*	*Patient information letter*
*Contact with community managers*	*Safety measures*
Chat, forum and diary	*Organizations involved*
	*Community managers*
***Additional functionalities (throughout the intervention when applicable)***	
Short videos (‘vlogs’) with survivors and professionals	
Quotes and stories from survivors	
Exercises	
*Tips for books, films, activities etc.*	

Note: The Minimal Intervention consists solely of the cursive components; the SAFE intervention consists of all mentioned components (cursive and non-cursive)

Community managers (CMs) manage the website and the registrations. Participants can use the website anonymously, for free, and as much as they want, without following a specific order as we take into account the differences in the individuals’ situations and needs. We do advise to start at the ‘Start here’ page where participants can find an explanation on how the website works. Furthermore, with a pop-up we advise on 24/7 help options in case of emergencies and on safely using the internet and SAFE.

CMs are (mental) health professionals or trainees supervised by (mental) health professionals. Participants can reach the CMs through e-mail, contact form, chat or forum. However, SAFE remains a self-support intervention, meaning the CMs do not initiate contact with participants, other than within the themed chats. Participants receive automated e-mails with a neutral name (“update from your menstrual calendar”), guiding them through the informed consent process and the questionnaires.

The website launched on April 1st 2019 and is locked during the RCT. Components that are still dynamic during the RCT are the chat, forum, pages with news and tips for books etc., and the help database. Bug fixes and downtimes will be registered.

### Outcome-measures

Participants are invited to complete surveys at three or four points in time: at registration (T0), at three months (T1), at six months (T2), and at 12 months (T3; only for participants in the first inclusion group). A Web Evaluation Questionnaire is completed after one month and at T2. The primary outcome is self-efficacy at six months, measured with the General Self Efficacy Scale (GSE). This scale assesses perceived self-efficacy in coping ability and adaptation to stressful life events [29, 30, 34]. We hypothesize that the intervention group will score a higher mean self-efficacy score than the comparison group at six months post-baseline.

The secondary outcomes are anxiety and depression, awareness, perceived social support, fear of partner, and perceived support from the website (Table 2).

**Table 2:** Secondary outcome measures

Outcome	Measure	Description	Hypothesis
Anxiety and depression	Hospital Anxiety and Depression Scale (HADS)	To determine levels of experienced anxiety and depression symptoms [35–37].	Participants in the intervention group will show a lower mean depression score and a lower mean anxiety score than the comparison group.
Awareness	Contemplation Ladder (modified version; original by Biener & Abrams [38])	To measure awareness of abuse from 0 to 10 based on how ready the woman is to make changes to her situation.	Participants in the intervention group will show a higher mean score on awareness than the comparison group.
Perceived social support	Medical Outcomes Survey – Social Support (MOS-SS5)	The questions concern the access to support of women to persons in their life [39, 40].	Participants in the intervention group will show a higher mean score on perceived social support than the comparison group.
Fear of partner	Visual Analogue Scale (VAS)	To measure the current level of fear of their (ex-) partner from 0 to 10.	Participants in the intervention group will show a lower mean fearfulness score than the comparison group.
Perceived website support	Visual Analogue Scale (VAS)	To measure how supported the participant feels by the website from 0 to 10.	Participants in the intervention group will show a higher mean score on perceived website support than the comparison group.

Other outcomes are general characteristics, measured by the General Characteristics Questionnaire (GCQ) and masculinity-femininity and gender roles, measured with the Bem Sex Role Inventory (BSRI) [41–43]. The impression and use of the intervention are assessed with the Web Evaluation Questionnaire (WEQ), including questions on relevance, language, lay-out, understandability, completeness, structure, findability and ease of use [44].

### Statistical analysis

Characteristics of the intervention and the comparison group will be assessed to study comparability of the groups. Missing data is expected to occur frequently in an internet-based self-support intervention because of loss-to-attrition. Missing data will be examined prior to analysis and the option of multiple imputation will be evaluated.

In the statistical analysis we will use an ANCOVA model and a Generalized Estimation Equation (GEE) model to analyze repeated measures. These models consider that the measures are clustered within a participant. The GEE model can test the difference in effect between the SAFE group and the Minimal Intervention group at different times of measuring.

As a sensitivity analysis, we will also perform complete case analyses. In both cases, we will use an ANCOVA analysis corrected for baseline to assess the intervention effect on short (T1) and long (T2 and T3) term. Following, we will look for the role of gender (derived from BSRI and General Characteristics data) as an outcomes modulator. Gender does not necessarily correlate with biological sex [45] and might be an independent predictor of outcomes [46]. A *p*-value of < 0.05 is considered statistically significant, based on two sided tests. Regardless of these formal significance levels, all results will be reported with corresponding *p* values to allow for situational judgment by the reader. All analysis will be performed is SPSS version 25.

A data management plan has been made which will be monitored independently to ensure safety of data and proper execution of the RCT. Personal information will be coded and the key to the code is only accessible for the project leader, the project coordinator and the research assistant. The quantitative data from questionnaires will be anonymized and other quantitative data as well as qualitative data will be pseudonymized. The data will be kept for 15 years.

### Safety and security

In building the intervention, numerous safety and security issues had to be considered, at hardware and software, user and provider levels. Firstly, the website runs with software that is updated according to the latest security and privacy requirements. Data from participants is encrypted and safely stored on a separate, protected server. Only indicated/selected members of the research team can access the data. Secondly, the website provides an escape button for participants to use when they have to exit the intervention immediately. Clicking on the escape button will close the intervention and open a neutral website. Also, tips with regard to safe internet use and erasing browser history are present. Thirdly, CMs all work according to a safety protocol, based on the national code on reporting domestic violence and child abuse (in Dutch: “*meldcode huiselijk geweld en kindermishandeling”* [47, 48]) in case of an emergency (e.g. a participant contacting us saying she is in immediate danger). CMs are available on weekdays from 08:00 till 17:00. With pop-ups on the website we make sure participants know we are not a help hotline, nor can they reach us 24/7, instead we refer them to services that can be reached 24/7. Furthermore, e-mails are sent with a neutral name, thus they are not immediately identifiable as messages from SAFE. All features of the intervention were approved by the Arnhem-Nijmegen medical ethics committee. In case of an adverse event, this will be recorded and reported to the medical ethics committee and the sponsor.

## Process evaluation methods and design

The process evaluation consists of several parts, with part one and two relying on data automatically collected from the website through surveys and website data, and part three consisting of a qualitative interview study among users.

First, we will evaluate the feasibility according to the following measures from Bowen and colleagues [49]: acceptability, demand, implementation, practicality, adaptation, integration, limited-efficacy testing. We will focus on intention to use, followed by an analysis of actual use and continued use. Quantitative data will be supported by qualitative information from the self-reported Web Evaluation Questionnaire (WEQ). Quantitative and qualitative measures of user satisfaction will help assess acceptability. Appropriateness will be evaluated comparing user wishes and needs, including safety, with expected goals as reported in the general questionnaire and WEQ. All quantitative data will be analyzed using mean differences between the intervention and control group. All qualitative data will be analyzed using a thematic coding approach [50, 51].

Secondly, we will perform a mixed-method analysis of online data from chat, mail and forum from the first 18 months after starting the RCT. Website data will be linked to participants’ characteristics using participant numbers and nicknames. To analyze the qualitative data we will primarily use an open thematic coding approach [50, 51]. Forum data will be chosen as the basis for qualitative analysis because of the wide range of subjects that we expect will be discussed on the forum. In addition to the thematic data approach, we will perform a word count in Atlas.ti (Scientific Software Development GmbH; Berlin, Germany) to analyze all text lines of online data.

Thirdly, we will analyze the experiences of the participants in semi-structured.

interviews. The interview guide will contain questions on how the participant first found SAFE, their experience of using it, features they liked and disliked, recommendations for improvements or changes, and their perceptions of how using the intervention had impacted on their mental health and safety decision-making and planning processes. Particular attention will be paid to how women maintained safety and confidentiality. All interviews will be tape-recorded and transcribed verbatim. Open thematic coding will be used to analyze the interviews [50, 51].

## Open feasibility study methods and design

The open feasibility study will take place 1 month after the RCT trial ends. The website will be open (no initial registration, informed consent and randomization procedure) for the public for a duration of 3 months. The lockdown of the intervention, safety measures and handling bug fixes are the same as in the RCT. The aim of the feasibility study is to create a real-world situation in which we can test the use of the intervention without the boundaries that an RCT trial poses for women wanting to use SAFE. Especially for this group, anonymity and easy access can be crucial for acceptability. The full intervention will be available and during the open feasibility study we monitor the attendance and usage of the website, e.g. how many people visit the website and what webpages do they visit. Women who want to use the chat and forum have to register (name, age, sex, experienced IPV (yes/no), reason for registering, email address, and password) in order to gain access. Community managers will monitor this process and activity on the website. The feasibility data from this study will be compared to the feasibility data from the RCT participants. Subsequently, this data will be used in further development and implementation of SAFE.

## Discussion

eHealth interventions for women exposed to IPV have the potential to break barriers in disclosing IPV to healthcare professionals and escape the unsafe environment. Therefore, we developed SAFE as a new means of help for these women. We will evaluate whether SAFE is an effective intervention to increase self-efficacy in women exposed to IPV, to increase awareness and perceived support, and to lower (mental) health symptoms, regarding depression and anxiety, in women exposed to IPV. Furthermore, we evaluate the feasibility of SAFE in a study and real-world setting.

However, there are some limitations to this study. For example, the women we aim to study are hard to reach and attrition rates in these types of studies are high. They might be hesitant to use SAFE out of fear of their partner. Also, the registration procedure and participating in a scientific study are potential barriers. Another challenge is promoting SAFE to the lay audiences, as we cannot disclose too much about the intervention due to differences in the intervention and control arm.

The study does, however, also have significant strengths compared to standard of care. Women can use SAFE anonymously and for free. Both arms of the intervention provide participants with significant information on IPV, safe relationships and help options. The intervention is based on scientific knowledge about IPV and eHealth, similar interventions in other countries [24–27], and on experiences and knowledge from female survivors and IPV experts. We do, therefore, provide a state-of-the-art intervention adapted to local specificities.

If SAFE proves to be a successful intervention, it could easily be implemented in the (mental) healthcare system as a national go-to spot for women exposed to IPV. Especially for those experiencing barriers in disclosing IPV and seeking help. Furthermore, it could be easily adapted and transferred to other European realities, as the help system is organized in a comparable manner across different countries. In conclusion, eHealth has the potential to reach many women who deal with IPV, while being receptive to their needs in particular situations and stages of change and encouraging them to reflect on their situation and seek professional help sooner.

## Data Availability

Not applicable.

## Abbreviations

CMs: Community managers
DV: Domestic violence
IPV: Intimate partner violence
PTSS: Post-traumatic stress syndrome
RCT: Randomized controlled trial
WEQ: Web Evaluation Questionnaire

## References

[1] WHO (2013). Global and regional estimates of violence against women: prevalence and health effects of intimate partner violence and non-partner sexual violence.

[2] Devries KM, Mak JY, Garcia-Moreno C, Petzold M, Child JC, Falder G (2013). Global health. The global prevalence of intimate partner violence against women. Science..

[3] Fra E. Violence against women: an EU-wide survey. Rep Eur Union Agency Fundam Rights. 2014:1–193.

[4] Ellsberg M, Jansen HA, Heise L, Watts CH, Garcia-Moreno C (2008). Intimate partner violence and women's physical and mental health in the WHO multi-country study on women's health and domestic violence: an observational study. Lancet.

[5] Rees S, Silove D, Chey T, Ivancic L, Steel Z, Creamer M (2011). Lifetime prevalence of gender-based violence in women and the relationship with mental disorders and psychosocial function. Jama..

[6] Attala JM, Bauza K, Pratt H, Vieira D (1995). Integrative review of effects on children of witnessing domestic violence. Issues Compr Pediatr Nurs.

[7] Carpenter GL, Stacks AM (2009). Developmental effects of exposure to intimate partner violence in early childhood: a review of the literature. Child Youth Serv Rev.

[8] Kitzmann KM, Gaylord NK, Holt AR, Kenny ED (2003). Child witnesses to domestic violence: a meta-analytic review. J Consult Clin Psychol.

[9] Ehrensaft MK, Cohen P, Brown J, Smailes E, Chen H, Johnson JG (2003). Intergenerational transmission of partner violence: a 20-year prospective study. J Consult Clin Psychol.

[10] Sox R (2004). Integrative review of recent Child witness to violence research. Clin Excell Nurse Pract.

[11] Widom CS, Czaja SJ, Dutton MA (2008). Childhood victimization and lifetime revictimization. Child Abuse Negl.

[12] Bair-Merritt MH, Lewis-O’Connor A, Goel S, Amato P, Ismailji T, Jelley M (2014). Primary care–based interventions for intimate partner violence: a systematic review. Am J Prev Med.

[13] Feder G, Ramsay J, Dunne D, Rose M, Arsene C, Norman R (2009). How far does screening women for domestic (partner) violence in different health-care settings meet criteria for a screening programme? Systematic reviews of nine UK National Screening Committee criteria.

[14] Feder GS, Hutson M, Ramsay J, Taket AR (2006). Women exposed to intimate partner violence: expectations and experiences when they encounter health care professionals: a meta-analysis of qualitative studies. Arch Intern Med.

[15] Hegarty KL, Taft AJ (2001). Overcoming the barriers to disclosure and inquiry of partner abuse for women attending general practice. Aust N Z J Public Health.

[16] O'Doherty LJ, Taft A, McNair R, Hegarty K (2016). Fractured identity in the context of intimate partner violence: barriers to and opportunities for seeking help in health settings. Violence Against Women.

[17] Ramsay J, Carter Y, Davidson L, Dunne D, Eldridge S, Feder G (2009). Advocacy interventions to reduce or eliminate violence and promote the physical and psychosocial well-being of women who experience intimate partner abuse. Cochrane Database Syst Rev.

[18] Reisenhofer S, Taft A (2013). Women's journey to safety - the transtheoretical model in clinical practice when working with women experiencing intimate partner violence: a scientific review and clinical guidance. Patient Educ Couns.

[19] Taft A, O'Doherty L, Hegarty K, Ramsay J, Davidson L, Feder G. Screening women for intimate partner violence in healthcare settings. Cochrane Database Syst Rev. 2013;(4):Cd007007. 10.1002/14651858.CD007007.pub2. 10.1002/14651858.CD007007.pub2 23633338

[20] Meier C, Fitzgerald M, Smith J. eHealth: extending, enhancing, and evolving health care. Annu Rev Biomed Eng. 2013;15:359–82. 10.1146/annurev-bioeng-071812-152350 23683088

[21] Schiavo R (2008). The rise of E-health: current trends and topics on online health communications. J Med Mark.

[22] Lv G-P, Kelders SM, Kip H, Sanderman R (2018). eHealth research, theory and development: a multidisciplinary approach.

[23] van Rosmalen-Nooijens KAWL, Lo Fo Wong SH, Prins JB, ALM L-J (2017). Young people, Adult worries: RCT and feasibility study of the internet-based self-support method “Feel the ViBe” for adolescents and young adults exposed to family violence. JMIR.

[24] Hegarty K, Tarzia L, Murray E, Valpied J, Humphreys C, Taft A (2015). Protocol for a randomised controlled trial of a web-based healthy relationship tool and safety decision aid for women experiencing domestic violence (I-DECIDE). BMC Public Health.

[25] Koziol-McLain J, Vandal AC, Wilson D, Nada-Raja S, Dobbs T, McLean C (2018). Efficacy of a web-based safety decision aid for women experiencing intimate partner violence: randomized controlled trial. J Med Internet Res.

[26] Eden KB, Perrin NA, Hanson GC, Messing JT, Bloom TL, Campbell JC (2015). Use of online safety decision aid by abused women: effect on decisional conflict in a randomized controlled trial. Am J Prev Med.

[27] Ford-Gilboe M, Varcoe C, Scott-Storey K, Wuest J, Case J, Currie LM (2017). A tailored online safety and health intervention for women experiencing intimate partner violence: the iCAN plan 4 safety randomized controlled trial protocol. BMC Public Health.

[28] Eysenbach G, Group C-E (2011). CONSORT-EHEALTH: improving and standardizing evaluation reports of web-based and mobile health interventions. J Med Internet Res.

[29] Schwarzer R, Jerusalem M (2010). The general self-efficacy scale (GSE). Anxiety Stress Coping.

[30] Teeuw B, Schwarzer R, Jerusalem M. Dutch adaptation of the general perceived self-efficacy scale. See: http://userpage. fu-berlin. de/~ health/dutch. htm; 1994.

[31] Hegarty K, Tarzia L, Murray E, Valpied J, Humphreys C, Taft A (2015). Protocol for a randomised controlled trial of a web-based healthy relationship tool and safety decision aid for women experiencing domestic violence (I-DECIDE). BMC Public Health.

[32] Koziol-McLain J, Vandal AC, Nada-Raja S, Wilson D, Glass N, Eden KB (2015). A web-based intervention for abused women: the New Zealand isafe randomised controlled trial protocol. BMC Public Health.

[33] Eysenbach G (2005). The law of attrition. J Med Internet Res.

[34] Rensen C, Bandyopadhyay S, Gopal PK, Van Brakel WH (2011). Measuring leprosy-related stigma–a pilot study to validate a toolkit of instruments. Disabil Rehabil.

[35] Bjelland I, Dahl AA, Haug TT, Neckelmann D (2002). The validity of the hospital anxiety and depression scale: an updated literature review. J Psychosom Res.

[36] Spinhoven P, Ormel J, Sloekers P, Kempen G, Speckens A, Van Hemert A (1997). A validation study of the hospital anxiety and depression scale (HADS) in different groups of Dutch subjects. Psychol Med.

[37] Zigmond AS, Snaith RP (1983). The hospital anxiety and depression scale. Acta Psychiatr Scand.

[38] Biener L, Abrams DB (1991). The contemplation ladder: validation of a measure of readiness to consider smoking cessation. Health Psychol.

[39] McCarrier K, Bushnell D, Martin M, Paczkowski R, Nelson D, Buesching D (2011). PRM16 validation and psychometric evaluation of a 5-item measure of perceived social support. Value Health.

[40] Sherbourne CD, Stewart AL (1991). The MOS social support survey. Soc Sci Med.

[41] Bem SL (1977). On the utility of alternative procedures for assessing psychological androgyny. J Consult Clin Psychol.

[42] Holt CL, Ellis JB (1998). Assessing the current validity of the Bem sex-role inventory. Sex Roles.

[43] Hoffman RM, Borders LD (2001). Twenty-five years after the Bem sex-role inventory: a reassessment and new issues regarding classification variability. Meas Eval Couns Dev.

[44] Elling S, Lentz L, de Jong M, Wimmer MA, Scholl J, Grönlund Å (2007). Website Evaluation Questionnaire: Development of a Research-Based Tool for Evaluating Informational Websites. Electronic Government: 6th International Conference, EGOV 2007, Regensburg, Germany, September 3–7, 2007 Proceedings.

[45] Tannenbaum C, Ellis RP, Eyssel F, Zou J, Schiebinger L (2019). Sex and gender analysis improves science and engineering. Nature..

[46] Pelletier R, Khan NA, Cox J, Daskalopoulou SS, Eisenberg MJ, Bacon SL (2016). Sex Versus Gender-Related Characteristics. Which Predicts Outcome After Acute Coronary Syndrome in the Young?. J Am Coll Cardiol.

[47] Ministerie van Justitie en Veiligheid MvV, Welzijn en Sport (2018). Basisdocument het afwegingskader in de meldcode huiselijk geweld en kindermishandeling.

[48] KNMG (2019). Nieuwe KNMG-meldcode kindermishandeling en huiselijk geweld per 1 januari 2019 verplicht.

[49] Bowen DJ, Kreuter M, Spring B, Cofta-Woerpel L, Linnan L, Weiner D (2009). How we design feasibility studies. Am J Prev Med.

[50] Ayres L (2014). Thematic Coding and Analysis. The SAGE Encyclopedia of Qualitative Research Methods. SAGE Publications, Inc.

[51] Boyatzis RE (1998). Transforming qualitative information: thematic analysis and code development: sage.

